# Oral Etoposide and Trastuzumab Use for HER2-Positive Metastatic Breast Cancer: A Retrospective Study from the Institut Curie Hospitals

**DOI:** 10.3390/cancers14092114

**Published:** 2022-04-24

**Authors:** Clelia Chalumeau, Matthieu Carton, Alexandre Eeckhoutte, Stelly Ballet, Anne Vincent-Salomon, Perrine Vuagnat, Audrey Bellesoeur, Jean-Yves Pierga, Marc-Henri Stern, Francois-Clement Bidard, Florence Lerebours

**Affiliations:** 1 Department of Medical Oncology, Institut Curie, 92210 St Cloud, France; vuagnatperrine@gmail.com (P.V.); francois-clement.bidard@curie.fr (F.-C.B.); florence.lerebours@curie.fr (F.L.); 2 Biostatistics, Institut Curie, 75005 Paris, France; matthieu.carton@curie.fr; 3 DNA Repair and Uveal Melanoma (D.R.U.M.), Inserm U830, Institut Curie, 75248 Paris, France; alexandre.eeckhoutte@curie.fr (A.E.); marc-henri.stern@curie.fr (M.-H.S.); 4 Institut Curie, PSL Research University, 75005 Paris, France; 5 Department of Diagnostic and Theranostic Medicine, Institut Curie, 75005 Paris, France; stelly.ballet@curie.fr (S.B.); anne.salomon@curie.fr (A.V.-S.); 6 Department of Medical Oncology, Institut Curie, 75005 Paris, France; audrey.bellesoeur@curie.fr (A.B.); jean-yves.pierga@curie.fr (J.-Y.P.); 7 Health Faculty, University of Paris, 75005 Paris, France; 8 UVSQ/Paris Saclay University, 78000 Versailles, France

**Keywords:** HER2 metastatic breast cancer, oral etoposide, trastuzumab, *TOP2A/ERBB2* co-amplification

## Abstract

**Simple Summary:**

Oral etoposide (VP16), an inhibitor of topoisomerase-II, has demonstrated clinical activity in metastatic breast cancer (MBC). To our knowledge, oral VP16 combined with trastuzumab (VP16-T) in HER2+ MBC has not been evaluated before. This combination is biologically relevant, as *TOP2A*, the gene encoding topoisomerase II, is often co-amplified with *ERBB2*. We report a retrospective analysis of the impact of oral VP16-trastuzumab on HER2+ MBC patients, together with *TOP2A/ERBB2* co-amplification status, assessed through shallow whole genome sequencing. In addition to its low cost and convenience, the oral VP16-trastuzumab regimen has shown a satisfactory activity and excellent tolerability.

**Abstract:**

Background: The *TOP2A* and *ERBB2* genes are co-amplified in about 40% of HER2 positive (HER2+) breast cancers. Oral etoposide (VP16), an inhibitor of topoisomerase-II (encoded by *TOP2A*), has demonstrated clinical activity in metastatic breast cancer (MBC). The benefit of oral VP16 combined with trastuzumab (VP16-T) in HER2+ MBC has not yet been evaluated. Methods: Patients treated at the Institut Curie Hospitals with VP16-T for HER2+ MBC were retrieved by an in silico search. Progression-free survival (PFS), overall survival (OS), response rate, prolonged PFS (defined as at least 6 months), clinical benefit, and toxicity were assessed. The co-amplification of *ERBB2* and *TOP2A* was assessed by shallow whole genome sequencing on tumor tissue whenever available. Results: Forty-three patients received VP16-T after a median number of six prior treatment lines for HER2+ MBC. Median PFS and OS were 2.9 months (95% CI [2.4–4.7]) and 11.3 months (95% CI [8.3–25.0]), respectively. Three patients had a complete response, while 12/40 (30%) experienced clinical benefit. Only three patients stopped treatment for toxicity. Seven (35%) patients displayed a *TOP2A/ERBB2* co-amplification. No statistically significant correlation was found between outcome and *TOP2A/ERBB2* co-amplification. Conclusion: Our analysis suggests a favorable efficacy and toxicity profile for VP16-T in patients with heavily pretreated HER2+ MBC.

## 1. Introduction

Approximately 15% of breast cancers display an amplification of *ERBB2*, which encodes the human epidermal growth factor receptor 2 (HER2) and is associated with poor prognosis [1,2,3]. HER2-targeted cancer therapies such as trastuzumab, pertuzumab, trastuzumab emtansine (T-DM1), lapatinib and newer therapies (such as trastuzumab deruxtecan and tucatinib) have significantly improved outcomes for HER2+ metastatic breast cancer (HER2+ MBC) patients [4,5,6,7,8,9]. Current treatment guidelines support the maintenance of anti-HER2 therapy throughout different lines of treatment [10,11].

Oral etoposide (VP16) is an inhibitor of topoisomerase II. Oral VP16 has demonstrated good clinical activity in heavily pre-treated patients with HER2-negative MBC compared to other active chemotherapies such as capecitabine, paclitaxel, eribulin, or anthracyclines [12]. Although not currently recommended in the MBC guidelines, the use of oral VP16 could be relevant in heavily pre-treated MBC, with the advantages of oral administration, low cost, and manageable toxicity. Moreover, while anthracyclines exhibit cardiac toxicity as do anti-HER2-targeted agents [13], oral VP16 has no reported cardiac toxicity, thus allowing for combination therapy.

*TOP2A*, the gene encoding topoisomerase II, is located on the long arm of chromosome 17 (17q21-22), close to *ERBB2* [14]. It has been reported that up to 40% of HER2+ breast cancers display a co-amplification of *TOP2A* and ERBB2 genes [15], which has been investigated as a predictive marker of anthracycline efficacy [15,16,17,18,19] in HER2+ breast cancers, with controversial results [20,21,22,23]. 

To the best of our knowledge, there are no studies evaluating the efficacy of oral VP16 in combination with trastuzumab, a combination used in our institution as a palliative, late-line therapy for HER2+ MBC patients. Here, we report a retrospective evaluation of the efficacy and safety of oral VP16 combined with trastuzumab in HER2+ MBC, and assess the predictive value of *TOP2A/ERBB2* co-amplification.

## 2. Materials and Methods

### 2.1. Patients and Clinical Data

The research project was submitted and approved by the Internal Research Committee of the Institut Curie (No. DATA200187). A waiver of informed consent was obtained because of the retrospective nature of the study. 

Patients treated with oral VP16 and trastuzumab were retrieved by an in silico search in the database of the Institut Curie Hospitals (Paris and Saint Cloud, France). Computerized medical files were then manually inspected by experienced medical oncologists. The inclusion criteria were: HER2+ MBC female patients treated with oral VP16 in combination with trastuzumab, regardless of the treatment line. HER2+ tumors were defined according to the 2018 American Society of Clinical Oncology/College of American Pathologists guidelines [3]. Trastuzumab could have been received prior to oral VP16 and continued after VP16-T treatment. All oral VP16 administration regimens were included in the study. A dose of 50 mg or 75 mg per day for 10–14 days out of 21 was defined as the standard oral VP16 regimen [24].

The primary objective was to evaluate the progression-free survival (PFS) in HER2+ MBC patients treated by VP16-T. PFS was defined as the period from initiation of combination therapy to disease progression or death for any cause, whichever came first. 

The secondary objectives were to evaluate overall survival (OS), progression-free survival (PFS) under the prior treatment line, response rate, clinical benefit, toxicity, and the predictive value of *TOP2/ERBB2* co-amplification. OS was calculated from the start of treatment until death from any cause, or until the last date the patient was known to be alive. The response rate was measured as the ratio of patients experiencing a partial or complete response using RECIST 1.1 criteria, considering patients who had measurable levels of disease at the treatment’s start [25]. Clinical benefit at 24 weeks was defined as a PFS > 24 weeks and/or objective tumor response. Toxicities were retrospectively classified according to the National Cancer Institute’s Common Criteria for Toxicity (version 5.0). 

### 2.2. TOP2A/ERBB2 Co-Amplification

*TOP2A/ERBB2* co-amplification was analyzed by shallow whole genome sequencing (sWGS) using formalin-fixed paraffin-embedded (FFPE) tumor tissue [26,27,28] from an available tumor tissue (from metastasis or the primary tumor). All slides were reviewed by a pathologist to ensure a minimum tumor cellularity of 30%. Between 5 and 50 ng (when available) of tumor deoxyribonucleic acid (DNA) were processed with the pre-capture kit XT-HS2 (Agilent) according to the manufacturing protocol. First, DNA samples were fragmented with the ME220 sonicator, reparated, adenylated and ligated with the duplex molecular barcode and the Illumina paired-end sequencing elements for 1 h. Then, unique dual sample indexes were added by 14 cycles of polymerase chain reaction (PCR) amplification. The libraries were qualified and quantified by the HS Qubit kit and TapeStation 4200 (Agilent) with the D1000 DNA ScreenTape analysis kit prior to pooling in one single tube. The final pool was finally quantified by quantitative PCR (qPCR) on the 7500 Real-Time PCR System (Thermo Fisher Scientific, Waltham, MA, USA). Then, 100 pb paired-end shallow sequencing was performed at the Institut Curie core sequencing facility using an Illumina Novaseq6000. 

Sequencing files were pre-processed as indicated in Eeckhoutte et al., 2020 [29]. Details are available upon request. Pre-processed alignment files were analyzed by counting and normalizing the number of aligned reads in a fixed window of 50 kb with quantitative DNA sequencing (QDNAseq) [30].QDNAseq associates contiguous windows considered to be in the same copy number level in genomic segments. The middle of the *TOP2A* and *ERBB2* loci were used to extract their respective fixed window and genomic segment values from the QDNAseq. QDNAseq outputs were then processed with shallow homologous recombination deficiency (shallowHRD) [29], which extracts a minimal copy number alteration (CNA) cut-off. 

The *TOP2A/ERBB2* co-amplification status was defined when the associated fixed window and segment values of both genes were 4-fold over the CNA cut-off. The absence of *TOP2A/ERBB2* co-amplification status was defined when the fixed window and segment values of *ERBB2* were 4-fold over the CNA cut-off, and those of *TOP2A* less than 4-fold of the CNA cut-off. Samples were classified as “not interpretable” in cases of discrepancies between the window and segment values for one gene, or if no amplification of *ERBB2* was retrieved by sWGS.

### 2.3. Statistics

Quantitative variables are presented with their median, minimum and maximum. Qualitative variables are presented with the number and percentage. Missing data (not available = NA) were excluded from the denominator for the calculation of percentages. Median follow-up was determined by the inverted Kaplan–Meier method [31]. Median values for PFS and OS (with their 95% confidence intervals [CI]) were estimated using the Kaplan–Meier method. All statistical analyses were performed using R 3.6 [32].

## 3. Results

### 3.1. Patients and Treatment

A number of 2003 patients treated for HER2+ MBC were retrieved by in silico screening of the Institut Curie electronic medical files. Among those patients, 43 met the inclusion criteria and were analyzed as part of this retrospective study: their characteristics are shown in Table 1. The median age of the diagnosis of primary breast cancer was 47 years (22–80 years). The median age of the diagnosis of MBC was 51 years (22–83 years). Synchronous BC metastases were diagnosed in 14 (33%) patients (de novo stage IV). The patients had received a median number of six prior treatment lines (range 0–12) at the time of receiving VP16-T regimen. Thirty-five patients (81%) had visceral metastases. The oral VP16 regimen was administered at the above-defined standard doses to 31 patients (72%). The median duration of VP16-T treatment was 2.9 months (0.2–14.6 months). VP16-T was stopped for disease progression (*n* = 35 patients, 81%), toxicity *(n* = 3 patients, 7%), therapeutic break (*n* = 3 patients, 7%) or unknown causes (*n* = 2 patients, 5%). 

### 3.2. Efficacy

**Figure 1:**
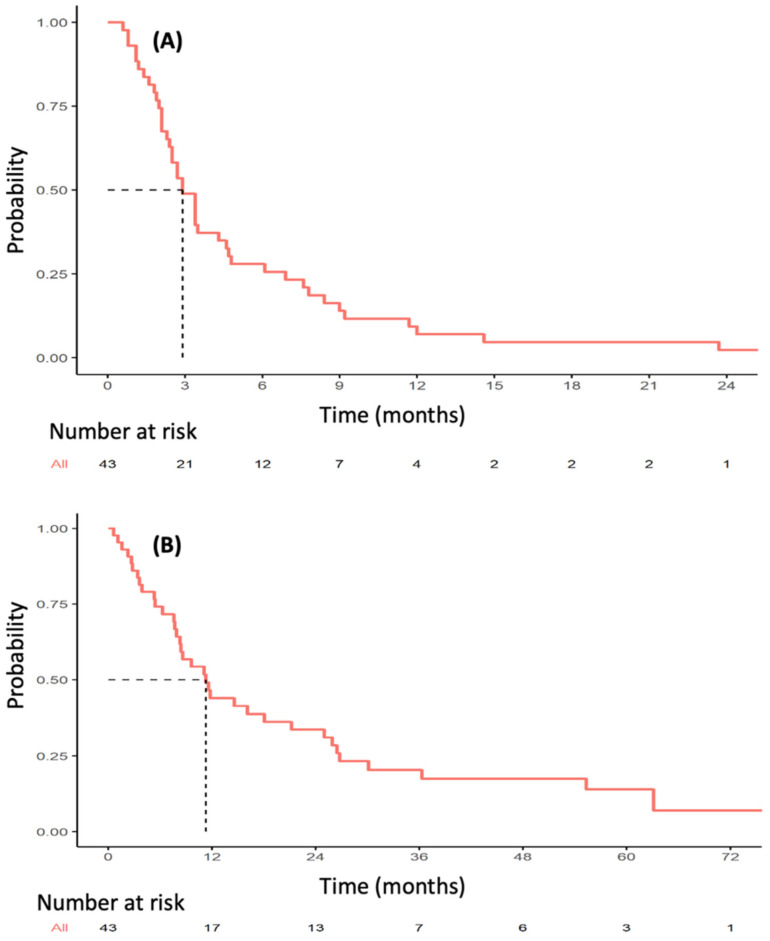
Kaplan–Meier estimates of progression-free survival (**A**) and overall survival (**B**) in patients treated with VP16-T.

**Figure 2:**
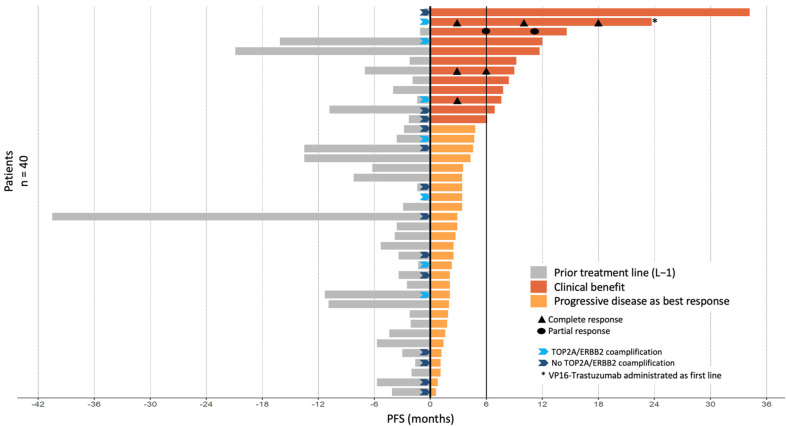
PFS by patient in relation to prior treatment lines and VP16-T. Clinical benefit was defined by either an objective tumor response (*n* = 4 patients) and/or a PFS under VP16-T for longer than 6 months (*n* = 8 patients).

The median follow-up was 56.8 months (range 3.8–82 months). Thirty-six PFS events were observed during VP16-T treatment. The median PFS was 2.9 months (95% CI [2.4–4.7]; Figure 1A). Median OS was 11.3 months (95% CI [8.3–25.0]) (Figure 1B). Forty patients were eligible for response rate assessment using RECIST 1.1 (Table S1). Four patients (10%) had a partial or complete response to VP16-T. A complete response was observed in three patients who received VP16-T as their first, second and thirteenth lines of treatment, respectively. One patient had a partial response. Overall, 12 out of 40 evaluable patients (30%) had a clinical benefit at 24 weeks (24 weeks clinical benefit rate: 30%; Figure 2).

The different systemic treatments administered immediately prior to VP16-T are detailed in Table S2 (one patient received VP16-T as a first-line treatment). Progression-free survival on prior treatment with gemcitabine-trastuzumab, vinorelbine-trastuzumab and cyclophosphamide-trastuzumab were 2.3 months (95% CI [2.2–NA]), 1.9 months (95% CI [0.8–NA]), and 3.4 months (95% CI [1.6–NA]), respectively. In 6 of the 12 patients with clinical benefit at 24 weeks, PFS with VP16-T was twice as long as the PFS under the prior line of treatment. Of note, the median number of prior treatment lines in these six patients was five (range 0–12), similar to the overall study population. All patients had previously received taxanes, and 63% had previously received anthracyclines. No significant differences in response rate or PFS were found between patients who had previously received anthracyclines or not.

Brain metastases were observed in 22 of 40 evaluable patients, and in 6 of 12 patients with prolonged PFS. Among these six patients with brain metastases and prolonged PFS, only one experienced a disease progression of her brain metastases while receiving VP16-T.

### 3.3. Toxicity

Toxicity was retrospectively assessed for the 42 patients (Table 2). Oral VP16 was discontinued due to toxicity in three patients: two for grade 3 nausea/vomiting, one for febrile neutropenia. Nauseas (grade 2 and 3) were observed in 14% of cases. Grade 1 alopecia was recorded in only one patient. No diarrhea, mucositis or allergies were observed. 

### 3.4. TOP2A/ERBB2 Co-Amplification

FFPE tumor samples were available for DNA extraction for 23 patients. sWGS was not interpretable for three samples. Among the 20 patients included in the sWGS analysis, 7 (35%) displayed an *TOP2A/ERBB2* co-amplification (examples are shown in Figure S1). Three patients with *TOP2A/ERBB2* co-amplification had a clinical benefit at 24 weeks (including two patients with complete response). The median PFS was 3.4 months (95% CI [2.3–6.9]) for these 20 cases, which is comparable to the overall study population (2.9 months, 95% CI [2.4–4.7]). No significant difference in median PFS in relation to the prior line was observed between the population with or without *TOP2A/ERBB2* co-amplification.

**Figure 3:**
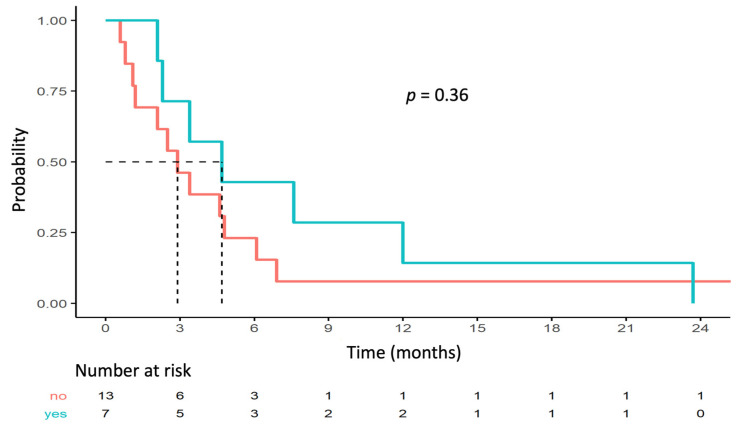
PFS depending on *TOP2A/ERBB2* co-amplification status.

No statistically significant correlation was found between outcome and *TOP2A/ERBB2* co-amplification. Median PFS rates for the populations with and without *TOP2A/ERBB2* co-amplification were 4.7 months (95% CI [2.3–NA]) and 2.9 months (95% CI [1.2–NA]; *p* = 0.36), respectively (Figure 3). Three (43%) patients with clinical benefit had *TOP2A/ERBB2* co-amplification and four (31%) patients without clinical benefit had *TOP2A/ERBB2* co-amplification (Fisher *p* = 0.65).

## 4. Discussion

To our knowledge, no studies have evaluated the efficacy of oral VP16 and trastuzumab combination in HER2+ MBC. We have shown that this combination achieves clinically meaningful PFS, with a prolonged PFS for a third of the patients (defined as PFS greater than or equal to 6 months), a clinical benefit in a third of the patients, and three complete responses. PFS and OS were 2.9 months and 11.3 months, respectively. These results were obtained in a heavily pre-treated population with a median number of six prior treatment lines for MBC. Moreover, most of our patient population displayed unfavorable clinical features, such as visceral metastases. The limitations of our study are related to its limited size and retrospective nature. However, this study is the first to specifically analyze the outcome and toxicity of oral VP16 associated with trastuzumab for HER2+ MBC.

Oral VP16 is a metronomic chemotherapy, defined as the regular administration of a minimally toxic dose of treatment over an extended period of time. In advanced breast cancer, metronomic chemotherapy has been shown to provide disease control with a lower incidence of adverse events compared to conventional chemotherapy at the maximum tolerated dose [33,34]. From 1994 to 2000, oral VP16 showed interesting clinical activity in patients with MBC after multiple lines of treatment [35,36,37,38,39]. More recently, a study by Cabel et al. [12] showed survival rates with oral VP16 comparable to other treatment lines including capecitabine, paclitaxel, eribulin, and anthracycline (median PFS of 3.2 months) in patients with HER2-negative MBC. 

Some studies reported the outcome of HER2+ MBC treated with oral VP16. In 2015, a retrospective study by Valaberga et al. [40] found a 4-month median PFS with oral VP16 in patients who had received a median of eight treatment lines (range 2–13). Twenty-one patients out of sixty-six had HER2+ MBC. The PFS did not differ between HER2-positive and HER2-negative status. Another retrospective study [41] included 110 pretreated patients with a median of 5 lines of treatment. Twenty-five of these patients had HER2+ MBC. The median duration of treatment was 4 months with, again, no significant difference according to HER2 status. In a prospective phase II study [42], a median PFS of 4.5 months was reported in 75 patients with MBC and a median number of 2 prior lines of therapy, of which 22 had an HER2+ disease. A review of twelve studies, of which HER2+ MBC patients comprised about a third, reported an overall 18.5% response rate with oral VP16 [43]. None of these studies specified the use of anti-HER2 therapy in combination with oral VP16. The low number of HER2+ MBC in these studies and the lack of specific subgroup analysis prevented any further comparison with our results.

There are limited data available on the efficacy of other late line chemotherapies and trastuzumab in pretreated HER2+ MBC. The efficacy of vinorelbine and trastuzumab was assessed in two prospective studies. In 46 patients treated with vinorelbine in a second-line setting after progression on a first-line taxane-based regimen, Blancas et al. [44] reported a 7-month median PFS in 7 HER2+ MBC patients. The phase II study of Lee et al. [45] showed a median PFS of 6.8 months in 33 HER2+ MBC patients with HER2+ MBC and a median of four prior lines of systemic treatment. Gemcitabine and trastuzumab have been investigated in two studies: Bartsch et al. [46] and Yardley et al. [47] included 23 and 37 patients, respectively. These studies included patients who received a median of two prior lines of systemic therapy for HER2+ MBC and reported a median PFS of 3 and 4 months, respectively. PFS rates of similar ranges were observed in the control arm of the TH3RESA trial [6]. In this pivotal trial, 602 HER2+ MBC patients who received a median of 4 prior lines of therapy demonstrated a significantly improved median PFS with trastuzumab-emtansine compared with physician-selected therapy (6.2 months versus 3.3 months). In the arm comprising the treatment of the physician’s choice, 68% of patients received concomitant trastuzumab and chemotherapy (vinorelbine in 32% of patients, gemcitabine in 16% of patients). Interestingly, the median PFS in the control arm of TH3RESA was similar to that observed with VP16 and trastuzumab in our report.

The presence of a co-amplification of *TOP2A and ERBB2* on chromosome 17 suggests a biological interest to combine oral VP16 and trastuzumab in HER2+ MBC. In keeping with prior reports, our sWGS analysis retrieved a *TOP2A/ERBB2* co-amplification in 35% of cases. *TOP2A/ERBB2* co-amplification was numerically, but not statistically, more frequent in patients benefiting from VP16-T. The limited number of patients analyzed prevents any definitive conclusion about the predictive value of the co-amplification. Of note, other non-genetic mechanisms may also modulate the response to topoisomerase 2 inhibitors [16], such as epigenetic mechanisms modulating DNA accessibility [48].

## 5. Conclusions

Finally, our retrospective study suggests oral VP16 and trastuzumab should be considered as a treatment option in heavily pre-treated HER2+ MBC patients. This combination yields prolonged responses in some patients, and has the advantages of oral administration, limited cost, and acceptable toxicity.

## Figures and Tables

**Table 1:** Patients’ characteristics.

		N Patients	%
Phenotype	HER2+	43	100
	HER2+/HR+	21	49
	HER2+/HR-	22	51
Age at primary BC (Years)	<50	27	63
	>50	16	37
Age at metastatic BC (Years)	<50	21	49
	>50	22	51
Stage at BC diagnosis	0 I II III IV	1 5 10 13 14	2 12 23 30 33
Histological type	DuctalLobular	38 5	88 12
Histological grade (EE)	1 2 3	3 19 21	7 44 49
Metastasis-Free Interval	de novo [6–24] months ]24–60] months >60 months	14 6 14 9	33 14 33 20
Number of metastatic sites	<2 >2	12 31	28 72
Visceral metastases	No Yes	8 35	19 81
Number of prior treatment lines	<2 3 4 5 6 7 >8	3 6 8 4 7 4 11	7 14 19 9 16 9 26
Median number of prior treatment lines	6 (0–12)	-	-
VP16 administration schedule	Standard * - 50 mg - 75 mg Other Not available	31 9 22 9 3	72 21 51 21 7

* 50–75 mg/D, 10 to 14D/21; HR—hormone receptor; EE—Elston and Ellis.

**Table 2:** Toxicities.

Toxicity	Grade 1 N (%)	Grade 2 N (%)	Grade 3 N (%)
Nausea	0	4 (10)	2 (5)
Neutropenia	3 (7)	1 (2)	1 (2)
Alopecia	1 (2)	0	0
Asthenia	17 (40)	10 (24)	8 (19)

Toxicity data were available for 42 patients.

## Data Availability

The data presented in this study are available on request from the corresponding author. The data are not publicly available due to ethical restrictions.

## Supplementary Materials

The following supporting information can be downloaded at: https://www.mdpi.com/article/10.3390/cancers14092114/s1, Table S1: Response rates under VP16-T; Table S2: Prior treatment before VP16-T; Figure S1: Example of *TOP2A/ERBB2* co-amplification.
