# Supplementary material for: Oral Etoposide and Trastuzumab Use for HER2-Positive Metastatic Breast Cancer: A Retrospective Study from the Institut Curie Hospitals
Source: Cancers (Basel). 2022 Apr 24;14(9):2114. doi: 10.3390/cancers14092114 (PMC9101021; doi:10.3390/cancers14092114)
Supplement: Supplementary file 1 [file cancers-14-02114-s001.zip › Figure S1.pdf]

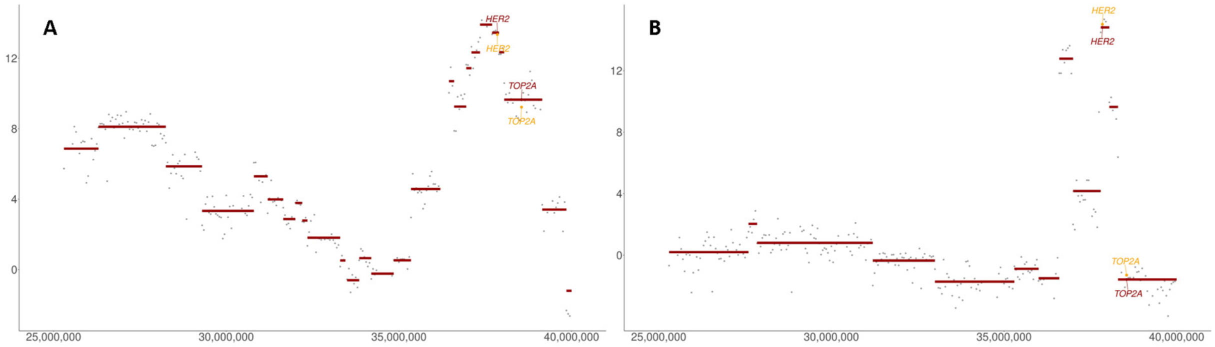

Figure S1: Example of TOP2A/ERBB2 co-amplification.

x-axis: genomic position on chromosome 17 (hg19); y-axis: number of CNA cut-off; grey dots: corrected read count for fixed windows of 50kb; red lines: segments associating contiguous windows with similar copy number status. Gene names in orange and red indicate their associated fixed window and genomic segment value, respectively. A. Example of TOP2A/ERBB2 co-amplification. B. Example of ERBB2 amplification without TOP2A co-amplification.
