# Supplementary material for: Oral Etoposide and Trastuzumab Use for HER2-Positive Metastatic Breast Cancer: A Retrospective Study from the Institut Curie Hospitals
Source: Cancers (Basel). 2022 Apr 24;14(9):2114. doi: 10.3390/cancers14092114 (PMC9101021; doi:10.3390/cancers14092114)
Supplement: Supplementary file 1 [file cancers-14-02114-s001.zip › Table S1 .pdf]

**Table S1:** Response rates under VP16-T. n=40 patients were evaluable for response rate**.**

| **Response by RECIST 1.1** | N patients (%) |
|---|---|
| Complete response | 3 (7.5) |
| Partial response | 1 (2.5) |
| Stable disease | 8 (20) |
| Progressive disease | 28 (70) |
