# Supplementary material for: Oral Etoposide and Trastuzumab Use for HER2-Positive Metastatic Breast Cancer: A Retrospective Study from the Institut Curie Hospitals
Source: Cancers (Basel). 2022 Apr 24;14(9):2114. doi: 10.3390/cancers14092114 (PMC9101021; doi:10.3390/cancers14092114)
Supplement: Supplementary file 1 [file cancers-14-02114-s001.zip › Table S2.pdf]

Table S2: Prior treatment before VP16-T. n=1 patient received VP16-T as first line therapy

**Prior treatment**	**n = 42**
gemcitabine + trastuzumab	7
vinorelbine + trastuzumab	6
cyclophosphamide + trastuzumab	5
lapatinib + trastuzumab	4
carboplatin + trastuzumab	4
trastuzumab-emtansine	4
capecitabine + lapatinib	3
taxane + trastuzumab	2
liposomal anthracycline	2
oral cyclophosphamide	2
other	3
